# Comparison of family-planning service quality reported by adolescents and young adult women in Mexico^☆^

**DOI:** 10.1016/j.ijgo.2015.12.003

**Published:** 2016-07

**Authors:** Blair G. Darney, Biani Saavedra-Avendano, Sandra G. Sosa-Rubi, Rafael Lozano, Maria I. Rodriguez

**Affiliations:** a Center for Health Systems Research, National Institute of Public Health Cuernavaca, Morelos, Mexico; b Oregon Health and Science University, Portland, OR, USA; c Institute for Health Metrics and Evaluation, University of Washington, Seattle, WA, USA

**Keywords:** Adolescent, Contraceptive services, Interpersonal quality, Family planning, Mexico, Quality, Technical quality

## Abstract

**Objective:**

Associations between age and patient-reported quality of family planning services were examined among young women in Mexico.

**Methods:**

A repeated cross-sectional analysis of survey data collected in 2006, 2009, and 2014 was performed. Data from women aged 15–29 years who had not undergone sterilization and were currently using a modern contraceptive method were included. The primary outcome was high-quality care, defined as positive responses to all five quality items regarding contraceptive services included in the survey. Multivariable logistic regression and marginal probabilities were used to compare adolescents and women aged 20–29 years. The responses of respondents using different contraceptive methods were compared.

**Results:**

Data were included from 15 835 individuals. The multivariable analysis demonstrated lower odds of reporting high-quality care among women aged 15–19 years (odds ratio 0.73; 95% confidence interval 0.60–0.88) and 20–24 years (odds ratio 0.85; 95% confidence interval 0.75–0.96) compared with women aged 25–29 years. Adolescents using hormonal and long-acting reversible contraception had significantly lower odds of reporting high-quality care compared with women aged 25–29.

**Conclusions:**

Adolescents in Mexico reported a lower quality of family planning services compared with young adult women. Continued research and policies are needed to improve the quality of contraceptive services.

## 1 Introduction

Unintended pregnancy is a public health problem globally, and the morbidity and mortality risks of unintended pregnancy are magnified in adolescents [1], [2]. Effective contraception is a key strategy in preventing unintended pregnancy and improving health outcomes in adolescents [3]. Evidence suggests that the quality of contraceptive services is linked to the uptake and continuation of contraceptive methods [4], [5]. WHO has identified the quality of services as a core health and human rights principle in the provision of contraceptive services [6].

Quality of health care has been defined as the extent to which healthcare services improve health outcomes consistent with current professional knowledge [7]. To be considered high quality, healthcare should be safe, effective, patient centered, timely, efficient, and accessible [7]. Frameworks for measuring the quality of family-planning services [6], [8], [9] draw on the broader healthcare literature, and emphasize both the technical and interpersonal domains of quality [10], [11]; technical quality is concerned with the application of evidence-based medicine and interpersonal quality focuses on provider–patient interactions [10].

Globally, significant disparities in contraceptive use persist despite overall declines in fertility and improvements in female education [12]. In Mexico, access to contraception is embedded in national policy [13]. However, despite large declines in fertility generally, adolescent fertility in Mexico has remained a persistent health problem [14], and contraceptive use remains low among adolescents, especially among adolescents in rural areas [15], [16].

Quality of care is increasingly recognized as an important element in improving health outcomes and fulfilling human-rights obligations [6]; however, discrepancies in service quality for adolescents are well documented [17]. To meet the reproductive-health needs of a growing global population of adolescents and young people, it is essential that these patients' perceptions of care quality are understood.

The aim of the present study was to test associations between age and patient-reported quality of family planning services among a population of patients in Mexico using modern contraceptives who had not undergone sterilization. It was hypothesized that adolescents would report lower care quality in comparison with women aged 20–29 years, and that the reported quality would vary based on the method of contraception used.

## 2 Materials and methods

A repeated cross-sectional study was performed between February 1 and August 31, 2015 using survey data collected in 2006, 2009, and 2014. The data were generated by the “Encuesta Nacional de la Dinámica Demográfica”, a nationally representative survey employing two-stage stratified probability sampling from Mexico's 31 states and Mexico City [18], [19], [20]. Each survey had a high response rate (85%, 89%, and 91%, respectively) and included household and reproductive-history modules [18], [19], [20]. The present study included women aged 15–29 years who had not undergone sterilization and who were currently using a modern contraceptive method (excluding withdrawal, rhythm, and traditional methods) that had been obtained from a healthcare facility. Outcome data were extracted from five survey questions regarding the quality of individual's family-planning visits; data on survey respondent characteristics were also retrieved. For the present study, the responses to these questions were grouped as technical or interpersonal measures of quality (Supplementary Material S1). The present study was approved by the Research Ethics Committee at the National Institute of Public Health, Cuernavaca, Mexico. Informed consent was waived for the present secondary analysis because it used de-identified, publically available data.

The primary outcome was if individuals reported receiving high-quality care, defined by individuals giving a “yes” response to all five quality items included in the present study. The secondary outcomes were positive responses to the three questions classified as technical-quality items and to the two questions classified as interpersonal-quality items. The key independent variable was age, grouped as 15–19 years, 20–24 years, and 25–29 years.

Current contraceptive methods were defined as long-acting reversible contraception (LARC), hormonal contraception (pills, patch, progestin injectable), and condoms. If individuals reported using two methods, they were classified according to the most effective method used (e.g. a person who reported using oral contraceptives and condoms would be classified as using hormonal contraception).

Several individual and household-level characteristics that could influence perceptions of quality and contraceptive choices were included as variables in the analysis. An “education gap” was calculated for each survey respondent; this was defined as the number of years, or fraction thereof, of schooling completed by an individual compared with the length of schooling that would be expected given the individual's age. This variable allows comparison between adolescents, who could still be in school, with adults who have completed their education. Women were classified as having ever been married (including divorced and widowed) or cohabited, or not. Other variables included whether respondents had reported any type of employment outside the home in the week prior to completing the survey, the number of live deliveries they had experienced, and whether they reported having health insurance. Health insurance was classified as being employment-based, public insurance for those in the informal sector (Seguro Popular), or no insurance. It was hypothesized that having health insurance would not be associated with any particular method of contraception because contraception is theoretically available to all women in Mexico regardless of insurance status; however, having health insurance could be related to other health-seeking behaviors. Finally, where individuals reported obtaining contraception was classified as being either a public, employment-based, or private facility.

Respondent households were classified according to whether the head of the household spoke an indigenous language, the preferred classification of indigenous ethnicity used by the Mexican government. The gender of the household head and the presence of large households (> 6 people) were included as markers of poverty. Additionally, a household-level wealth index was constructed using data for household characteristics and property using factor analysis; this index was collapsed into quintiles. Whether households were in a rural location (< 2500 inhabitants) or not was recorded, and indicator variables for the 31 Mexican states and Mexico City were included. Finally, an indicator for survey year (2006, 2009, and 2014) was included in the analyses.

Descriptive statistics and bivariate tests (χ^2^ test or Student *t* test) were used to examine differences in outcomes and covariates between respondent age groups, and to describe trends in outcomes across survey years and age groups. Multivariable logistic regression models including all the study variables were used to examine the relationship between age and if respondents received high-quality care. Following this, a model was developed that was stratified by the contraceptive method used (separate models for LARC, hormonal contraception, and condoms); this model was applied to investigate associations between respondent variables and if respondents reported receiving high-quality care. Several sensitivity analyses were performed, examining interactions between potential effect modifiers and respondent age. The household-level wealth index variable contained considerable missing data (8.4% of values missing) (Supplementary Material S2); consequently, models were constructed without this variable to test the robustness of the models to the missing data.

Survey weights were used to account for the complex survey design and to produce population estimates. Marginal effects were calculated to simplify data interpretation (multivariable estimates of changes in probability that accounted for covariates). In the bivariate analysis, *P* < 0.05 was considered significant. In the multivariable analyses, odds ratios (ORs) not including 1.0 in the 95% confidence interval (CI), and predicted or marginal probabilities without overlapping CIs were considered significant. Stata 13.0 (StataCorp LP, College Station, TX, USA) was used for all analyses.

## 3 Results

The present study sample included 15 835 women aged 15–29 years who reported using a modern contraceptive that had been obtained at a healthcare facility. Among the complete estimated study population (N = 6 082 045), 13% were aged 15–19 years old, 42% were aged 20–24 years, and 45% were aged 25-29 years (Table 1). In comparison with women aged 20–24 and 25–29, fewer adolescents reported having ever been married or cohabited, and adolescents reported fewer previous live deliveries. Additionally, a larger proportion of adolescents reported having Seguro Popular insurance (Table 1).

When examining respondents' contraceptive use, in comparison with women aged 20–24 and 25–29, significantly more adolescents reported exclusive condom use. A lower proportion of adolescents reported LARC use in comparison with older individuals; however, this difference was not statistically significant. The overall difference in contraceptive methods used reported between the age groups was significant (*P* = 0.03) (Table 1). The proportion of adolescents who reported obtaining contraception at public healthcare facilities was significantly higher than in each of the 20–24-years and 25–29-years age groups (Table 1).

**Fig. 1:**
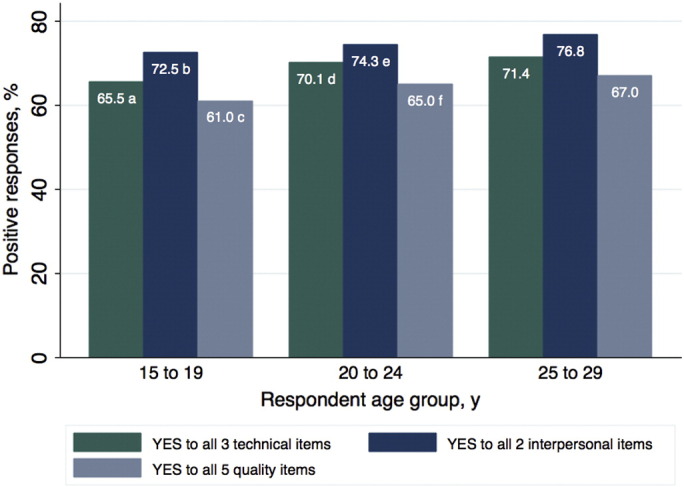
Positive responses to contraceptive healthcare quality items. Comparisons made using χ^2^ test (Ref. 25-29-y age group): ^a^*P* = 0.001; ^b^ Comparison with 25–29-y group (χ^2^) *P* = 0.001; ^c^ Comparison with 25–29-y group (χ^2^) *P* < 0.001; ^d^ Comparison with 25–29-y group (χ^2^) *P* = 0.097; ^e^ Comparison with 25–29-y group (χ^2^) *P* = 0.020; ^f^ Comparison with 25–29-y group (χ^2^) *P* = 0.033.

**Fig. 2:**
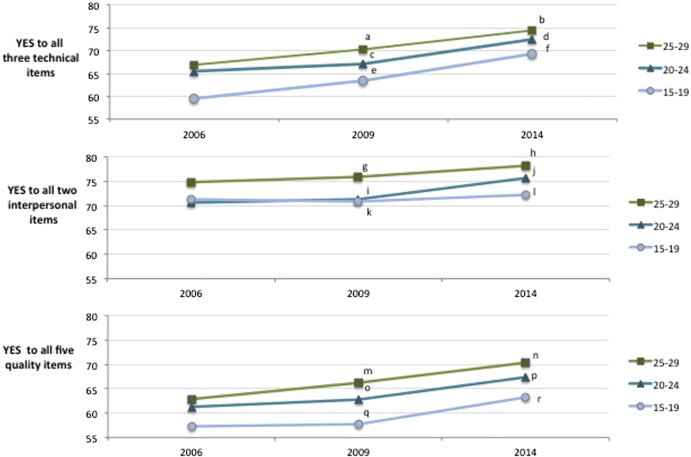
Positive responses to contraceptive healthcare quality items at each of the three survey years. Comparisons made using χ^2^ test (Ref. 2006): ^a^*P* = 0.114; ^b^*P* < 0.001; ^c^*P* = 0.518; ^d^*P* = 0.003; ^e^*P* = 407; ^f^*P* = 0.023; ^g^*P* = 0.567; ^h^*P* = 0.071; ^i^*P* = 0.780; ^j^*P* = 0.032; ^k^*P* = 0.928; ^l^*P* = 0.815; ^m^*P* = 0.140; ^n^*P* = 0.001; ^o^*P* = 0.588; ^p^*P* = 0.013; ^q^*P* = 0.935; ^r^*P* = 0.170.

The primary outcome, positive responses to all five quality items, was recorded for 61.0% of adolescents, 65.0% of women aged 20–24 years, and 67.0% of women aged 25–29 years; significant differences with the 25–29 year age group were observed for both adolescents (*P* < 0.001) and women aged 20–24 years (*P* = 0.03) (Fig. 1). Across the complete study population, the individual quality item reported by the fewest respondents was, “I was given enough time for all the information needed” (Table 1). The quality item, “possible side effects were explained”, was reported by significantly fewer adolescents compared with women aged 25–29 years. In all three age groups included in the study, the proportion of positive answers to all five quality items, the three technical items, and the two interpersonal items increased over the time points of the three surveys (Fig. 2). In all age groups, increases in the proportion of positive answers to all five quality items was driven by increases in positive responses to the three technical items; the proportion of adolescents responding positively to all three technical items increased from 59.5% to 69.2% between 2006 and 2014 (*P* = 0.02).

In the multivariable analyses, individuals aged 15–19 years (OR 0.73; 95%CI 0.60–0.88) and 20–24 years (OR 0.85; 95%CI 0.75–0.96), were less likely to answer positively to all five quality items in comparison with women who were aged 25–29 years (Table 2). This corresponded to an 8% reduction in the multivariable probability of positive responses to all five quality items among adolescents in comparison with women aged 25–29 years (assuming the means of all other covariates were maintained). Covariates that were significantly associated with positive responses to all quality items were individuals receiving the method of contraception they requested compared with those who did not, and being in the highest household wealth quintile compared with the poorest. Women with a larger educational gaps or living in indigenous households demonstrated lower odds of responding positively to all quality items. The ORs of responding positively were very similar when considering the technical and interpersonal items separately (Table 2).

Similar results were demonstrated in the sensitivity analyses when the household-level wealth variable was not included in the model to reduce the number of observations excluded from the multivariable models (data not shown). No evidence of effect modification (non-significant interactions, data not shown) was found and so effect modifiers were not considered further.

When the model was stratified by the contraceptive method used (Table 3), the associations observed remained consistent with those produced by the pooled data model. Adolescents using hormonal contraception were significantly less likely to respond positively to all five quality items compared with women aged 25–29 years using hormonal contraception. This corresponded to a 9% reduction in the multivariable probability of positive answers across all five quality items for adolescents using hormonal contraception in comparison with women aged 25–29 years using hormonal contraception (assuming the means of all other covariates were maintained). Both adolescents and women aged 20–24 years who were using LARC demonstrated lower odds of receiving high-quality care in comparison with women aged 25–29 years who were using LARC. Across all individuals who were using LARC included in the present study, larger odds of responding positively to all five quality items were recorded in the 2014 survey data compared with the 2006 survey data (Table 3).

## 4 Discussion

The present study identified reduced patient-reported quality of care for family planning among adolescents in comparison with older young women. Only 61.0% of adolescents responded positively to all five quality items, compared with 65.0% of women aged 20–24 years and 67.0% of women aged 25–29 years. Additionally, in comparison with women aged 25–29 years, both adolescents (15–19 years) and women aged 20–24 years who were currently using modern contraception that had been obtained at a healthcare facility were less likely to report receiving high-quality care. The results of the study suggest that self-reported care quality has increased for all three age groups between 2006 and 2014. Additionally, the study demonstrated that adolescents who were using hormonal contraception were significantly less likely to respond positively to all five quality items compared with women aged 25–29 years who were using hormonal contraception. Specifically, more adolescents reported not having their concerns about side effects addressed compared with women aged 25–29 years. These findings suggest that gaps exist in the quality of care received by adolescents and older women.

Very limited data exist regarding associations between age and differences in the quality of reproductive healthcare services [21]. Quality of healthcare is a concept that has multiple dimensions [6], [7], [11]. The Bruce-Jain framework [8] has been used to measure care quality in family planning programs for a long time; however, new guidance from WHO highlights the need to include further elements including the perspectives of individual patients [6]. The key weakness of patient-reported quality measures is that patients' memories and assessments of quality may not always be accurate, especially regarding technical quality. However, individuals are best placed to judge their own experiences of care. Comprehensive quality assessments are needed that rely on data from providers and patients, including observations and assessments of structures, processes, and health outcomes [22]. Valid and robust measures of patient-reported quality in reproductive healthcare are needed that both reflect local context and permit comparisons across studies and populations.

Low- and middle-income countries have placed significant emphasis on readiness to provide reproductive healthcare services; however, the implementation of guidelines and other evidence-based care that could improve technical and interpersonal quality has lagged behind [23]. In addition, changes in policy do not always translate into improvements in care quality [24]. By way of example, access to contraception, and the topic of adolescent pregnancy in particular, have been high on the policy agenda in Mexico [13], [25]; however, although per-capita spending on family planning has increased dramatically (from 59 Mexican Pesos in 2009 to 150 Pesos in 2012 [Personal communication: Ileana Heredia-Pi, Instituto Nacional de Salud Publica, November, 2015]), it remains below the guideline levels (US$ 16 in 2003 [26]). The present study provides evidence that quality of care could be improving over time, and it is essential that outcomes and patient-reported quality continue to be monitored. This is especially important in the public sector, as investment in family planning in Mexico increases.

The present study had limitations that are common to all observational studies. It is unknown how much time passed between respondents receiving contraceptives and responding to surveys; this could lead to recall bias. This is one of the reasons women older than 29 and individuals who had undergone sterilization were not included; it was considered likely that the individuals could have received contraceptives a considerable time before responding to surveys. Further, the present study only included individuals who were currently using contraception. Data were not included regarding individuals who had sought care but were not using contraception; these individuals could have reported lower quality than respondents who were using contraception. Consequently, the present study cannot provide information regarding the relationship between care quality and the uptake or continuation of contraception, which has been the focus of previous research [4], [5], [27]. However, the present study's focus on patient experiences of quality is consistent with current thinking about care quality in a human rights framework [6]. Finally, data were not collected for adolescents younger than 15 years of age, an important and vulnerable group.

Lessons learned from experience in Mexico can be used to guide other low- and middle-income countries, which continue to demonstrate high adolescent fertility despite policy and programmatic efforts to counter this. In the present study, adolescents were more likely to report low-quality care than women aged 20–24 years and 25–29 years. Continued effort is needed to measure and improve the quality of contraceptive services for all women; the results of the present study suggest there is additional work to be done to meet the needs of adolescents, both to ensure human rights and to prevent unintended pregnancies.

The following are the supplementary data related to this article.

Supplementary material S1 Patient-reported items of the quality of familyplanning services items. Supplementary material S1

Supplementary material S2 Missing survey data. Supplementary material S2

## Figures and Tables

**Table 1:** Sociodemographic and contraception characteristics of female survey respondents aged 15–29 years using modern contraception obtained from a healthcare facility (n = 15 835; N = 6 082 045). a

Variable	Sample survey respondents				*P* value
	Complete sample (n = 15 835; N = 6 082 045)	Individuals aged 15–19 y (n = 2307; N = 814 994)	Individuals aged 20–24 y (n = 6612; N = 2 554 458)	Individuals aged 25–29 y (n = 6916; N = 2 736 920)	
Educational gap, y b^,c^	0.91 (0.86–0.95)	0.93 (0.82–1.03)	0.76 (0.70–0.83)	1.04 (0.96–1.11)	0.002
Respondents have ever been married or co-habited	90.3 (89.6–91.0)	86.0 (83.8–88.0)	89.5 (88.3–90.6)	92.4 (91.4–93.4)	< 0.001
Currently working	31.3 (30.2–32.4)	17.1 (15.1–19.4)	30.2 (28.5–31.9)	36.7 (35.0–38.4)	< 0.001
Previous number of live deliveries c	1.65 (1.63–1.67)	1.18 (1.14–1.22)	1.49 (1.47–1.52)	1.94 (1.90–1.97)	< 0.001
Reported ever being pregnant	93.6 (93.0–94.1)	88.7 (86.6–90.4)	93.2 (92.2–94.1)	95.4 (94.7–96.1)	< 0.001
Health insurance					0.001
None	30.3 (29.0–31.6)	33.1 (30.1–36.1)	31.1 (29.2–33.1)	28.7 (26.9–30.4)	
Employment based	34.9 (33.7–36.1)	20.1 (17.8–22.5)	33.8 (32.0–35.6)	40.4 (38.6–42.2)	
Seguro Popular	34.8 (33.6–36.1)	46.9 (43.8–49.9)	35.1 (33.4–36.9)	31.0 (29.4–32.6)	
					
Household characteristics					
Head of household speaks an indigenous language	7.1 (6.4–7.8)	7.7 (6.3–9.4)	7.3 (6.4–8.3)	6.6 (5.8–7.6)	0.119
Male head of household	81.3 (80.3–82.2)	76.9 (74.2–79.4)	81.1 (79.6–82.5)	82.8 (81.4–84.1)	< 0.001
Household size ≥ 6 people	30.9 (29.7–32.1)	41.4 (38.4–44.4)	33.2 (31.4–35.0)	25.6 (24.0–27.3)	< 0.001
Household wealth quintile					< 0.001
1 (Poorest)	24.3 (23.3–25.4)	27.7 (24.9–30.6)	24.5 (23.0–26.1)	23.1 (21.7–24.7)	
2	24.9 (23.8–26.0)	26.1 (23.4–29.0)	25.2 (23.6–27.0)	24.2 (22.7–25.8)	
3	21.7 (20.7–22.8)	21.2 (18.8–23.8)	22.0 (20.4–23.7)	21.6 (20.2–23.2)	
4	17.8 (16.8–18.9)	16.2 (13.9–18.8)	16.9 (15.4–18.5)	19.1 (17.7–20.7)	
5 (Wealthiest)	11.3 (10.5–12.1)	8.8 (7.2–10.8)	11.4 (10.2–12.6)	11.9 (10.9–13.1)	
Rural locality (population ≤ 2500)	24.8 (23.7–26.0)	28.9 (26.3–31.7)	25.2 (23.6–26.9)	23.2 (21.8–24.6)	< 0.001
Contraceptive					0.033
Hormonal	22.3 (21.3–23.3)	21.1 (18.8–23.5)	22.3 (20.8–23.8)	22.7 (21.3–24.2)	
Long-acting reversible contraction	67.5 (66.3–68.5)	65.2 (62.3–68.0)	67.7 (66.0–69.4)	67.9 (66.3–69.5)	
Condoms	10.2 (9.6–11.0)	13.8 (11.8–16.0)	10.0 (9.1–11.1)	9.4 (8.4–10.5)	
Source of contraception					< 0.001
Employment-based facility	35.7 (34.6–36.9)	23.2 (20.8–25.7)	35.2 (33.5–37.0)	40.0 (38.2–41.7)	
Public facility	53.7 (52.5–54.9)	68.6 (65.7–71.3)	55.0 (53.2–56.9)	47.9 (46.1–49.7)	
Private facility	10.6 (9.8–11.4)	8.2 (6.5–10.4)	9.7 (8.6–11.0)	12.1 (10.9–13.4)	
Individual received their requested method of contraception	93.8 (93.2–94.3)	91.3 (89.5–92.9)	93.3 (92.3–94.1)	95.1 (94.4–95.7)	< 0.001
*Technical quality items*					
Individual received information on other contraceptives	81.0 (80.0–82.0)	79.2 (76.5–81.6)	81.2 (79.7–82.7)	81.3 (79.9–82.7)	0.230
Possible adverse effects were explained	78.2 (77.2–79.3)	74.2 (71.5–76.8)	78.1 (76.4–79.6)	79.6 (78.2–81.0)	0.001
Individual was told to return if adverse effects occurred	85.8 (85.0–86.6)	82.3 (79.8–84.6)	85.9 (84.6–87.1)	86.8 (85.6–88.0)	0.002
*Interpersonal quality items*					
Individual reported being given sufficient time for all the information needed	76.3 (75.2–77.2)	74.0 (71.2–76.6)	75.1 (73.4–76.8)	78.0 (76.5–79.4)	0.003
All an individual's doubts about the contraceptive were addressed	78.8 (77.8–79.7)	76.3 (73.6–78.8)	77.7 (76.2–79.2)	80.5 (79.1–81.8)	0.001

a Values are given as weighted percentage of respondents (95% confidence interval) unless indicated otherwise.

b Defined as the number of years of schooling completed by an individual expressed as a fraction of the number of years of completed schooling that would be expected given the individual's age.

c Values given as mean (95% confidence interval).

**Table 2:** Multivariable analysis of associations between the age of survey respondents and the reported quality of contraceptive services (n = 13 297; N = 5 056 173). a, b

Variable	Positive answers to all five quality measures	Positive answers to all three technical quality measures	Positive answers to both interpersonal quality measures
Age, y			
15–19	0.73 (0.60–0.88)	0.77 (0.63–0.94)	0.78 (0.64–0.96)
20–24	0.85 (0.75–0.96)	0.88 (0.77–0.99)	0.82 (0.72–0.94)
25–29	Ref.	Ref.	Ref.
Education gap, y	0.91 (0.88–0.94)	0.92 (0.88–0.95)	0.93 (0.90–0.96)
Respondents have ever been married or co-habited			
Yes	0.95 (0.75–1.20)	0.98 (0.77–1.24)	0.97 (0.76–1.25)
No	Ref.	Ref.	Ref.
Currently working			
Yes	1.03 (0.90–1.16)	1.05 (0.92–1.20)	1.02 (0.88–1.17)
No	Ref.	Ref.	Ref.
No. of previous live deliveries	0.98 (0.91–1.05)	0.99 (0.91–1.06)	0.98 (0.91–1.06)
Individual received their requested method of contraception			
Yes	3.00 (2.41–3.73)	3.21 (2.58–3.98)	2.79 (2.25–3.45)
No	Ref.	Ref.	Ref.
Health insurance			
Employment based	0.90 (0.77–1.04)	0.92 (0.79–1.07)	0.91 (0.77–1.07)
None	1.10 (0.94–1.29)	1.17 (0.99–1.38)	1.03 (0.86–1.22)
Seguro Popular	Ref.	Ref.	Ref.
Source of contraception			
Employment-based facility	1.05 (0.90–1.22)	1.08 (0.92–1.26)	1.06 (0.90–1.23)
Private facility	1.48 (1.15–1.90)	1.52 (1.15–1.99)	1.88 (1.38–2.55)
Public facility	Ref.	Ref.	Ref.
Head of household speaks an indigenous language			
Yes	0.78 (0.61–0.99)	0.76 (0.59–0.97)	0.85 (0.681–1.06)
No	Ref.	Ref.	Ref.
Male head of household			
Yes	0.98 (0.84–1.14)	1.00 (0.84–1.17)	1.04 (0.87–1.23)
No	Ref.	Ref.	Ref.
Household size ≥ 6 people			
Yes	1.00 (0.88–1.13)	1.01 (0.88–1.14)	0.97 (0.85–1.10)
No	Ref.	Ref.	Ref.
Household wealth quintile			
1 (Poorest)	Ref.	Ref.	Ref.
2	1.11 (0.95–1.30)	1.15 (0.98–1.35)	1.18 (0.99–1.39)
3	1.26 (1.05–1.51)	1.35 (1.11–1.62)	1.23 (1.01–1.49)
4	1.50 (1.22–1.84)	1.51 (1.21–1.87)	1.51 (1.19–1.90)
5 (Wealthiest)	1.48 (1.16–1.87)	1.44 (1.12–1.84)	1.43 (1.10–1.85)
Rural locality (population ≤ 2500)			
Yes	1.21 (1.06–1.37)	1.21 (1.05–1.38)	1.17 (1.017–1.34)
No	Ref.	Ref.	Ref.
Survey year			
2006	Ref.	Ref.	Ref.
2009	1.05 (0.89–1.24)	1.12 (0.94–1.32)	1.04 (0.87–1.23)
2014	1.24 (1.04–1.47)	1.37 (1.14–1.63)	1.20 (1.00–1.44)

a Values are given as odds ratios (95% confidence interval).

b The models used were adjusted according to the state that was the source of the data; consequently, 32 dummy variables were included.

**Table 3:** Multivariable analysis of associations between positive response to all five quality items and different contraceptives used (n = 13 247; N = 5 016 835). a, b

Variable	Hormonal contraception	Long-acting reversible contraception	Condoms
Age, y			
15–19	0.60 (0.41–0.88)	0.72 (0.57–0.91)	1.08 (0.59–1.95)
20–24	0.98 (0.74–1.27)	0.79 (0.67–0.91)	1.02 (0.70–1.47)
25–29	Ref.	Ref.	Ref.
Education gap, y	0.89 (0.84–0.95)	0.92 (0.88–0.95)	0.88 (0.79–0.96)
Respondents have ever been married or co-habited			
Yes	1.04 (0.56–1.92)	0.92 (0.71–1.19)	1.96 (0.70–5.45)
No	Ref.	Ref.	Ref.
Currently working			
Yes	1.24 (0.91–1.66)	0.96 (0.81–1.11)	0.96 (0.63–1.46)
No	Ref.	Ref.	Ref.
No. of previous live deliveries	0.88 (0.75–1.01)	1.01 (0.91–1.10)	1.04 (0.83–1.28)
Individual received their requested method of contraception			
Yes	2.65 (1.67–4.19)	3.69 (2.82–4.81)	1.20 (0.62–2.30)
No	Ref.	Ref.	Ref.
Health insurance			
Employment based	0.98 (0.70–1.35)	0.86 (0.70–1.03)	0.95 (0.59–1.53)
None	1.14 (0.78–1.67)	1.20 (0.98–1.45)	0.84 (0.52–1.35)
Seguro Popular	Ref.	Ref.	Ref.
Source of contraception			
Employment-based facility	0.81 (0.55–1.17)	1.11 (0.93–1.32)	1.20 (0.74–1.92)
Private facility	1.01 (0.60–1.67)	1.65 (1.22–2.21)	0.50 (0.13–1.83)
Public facility	Ref.	Ref.	Ref.
Head of household speaks an indigenous language			
Yes	0.58 (0.35–0.94)	0.81 (0.62–1.05)	1.03 (0.54–1.96)
No	Ref.	Ref.	Ref.
Male head of household			
Yes	1.03 (0.72–1.44)	0.97 (0.81–1.16)	0.79 (0.48–1.27)
No	Ref.	Ref.	Ref.
Household size ≥ 6 people			
Yes	0.85 (0.65–1.12)	1.06 (0.91–1.22)	1.21 (0.80–1.81)
No	Ref.	Ref.	Ref.
Household wealth quintile			
1 (Poorest)	Ref.	Ref.	Ref.
2	0.95 (0.68–1.32)	1.15 (0.94–1.39)	1.27 (0.81–1.96)
3	1.12 (0.74–1.69)	1.34 (1.08–1.65)	1.26 (0.74–2.11)
4	1.63 (0.98–2.70)	1.49 (1.16–1.90)	1.86 (0.97–3.55)
5 (Wealthiest)	2.72 (1.45–5.11)	1.33 (1.01–1.73)	3.18 (1.41–7.17)
Rural locality (population ≤ 2500)			
Yes	0.95 (0.74–1.20)	1.32 (1.12–1.55)	1.44 (0.99–2.08)
No	Ref.	Ref.	Ref.
Survey year			
2006	Ref.	Ref.	Ref.
2009	0.91 (0.65–1.25)	1.13 (0.91–1.39)	0.91 (0.57–1.45)
2014	1.07 (0.75–1.52)	1.35 (1.09–1.67)	1.02 (0.62–1.65)

a Values are given as odds ratios (95% confidence interval).

b The models used were adjusted according to the state that was the source of the data; consequently, 32 dummy variables were included.

## References

[1] Chandra-Mouli V., Camacho A.V., Michaud P.A. (2013). WHO guidelines on preventing early pregnancy and poor reproductive outcomes among adolescents in developing countries. J Adolesc Health.

[2] Conde-Agudelo A., Belizán J.M., Lammers C. (2005). Maternal-perinatal morbidity and mortality associated with adolescent pregnancy in Latin America: Cross-sectional study. Am J Obstet Gynecol.

[3] Baldwin M.K., Edelman A.B. (2013). The effect of long-acting reversible contraception on rapid repeat pregnancy in adolescents: a review. J Adolesc Health.

[4] Jain A.K., Ramarao S., Kim J., Costello M. (2012). Evaluation of an intervention to improve quality of care in family planning programme in the Philippines. J Biosoc Sci.

[5] RamaRao S., Lacuesta M., Costello M., Pangolibay B., Jones H. (2003). The link between quality of care and contraceptive use. Int Fam Plan Perspect.

[6] World Health Organization (2014). Ensuring human rights in the provision of contraceptive information and services: Guidance and recommendations.

[7] Committee on Quality of Health Care in America (2001). Institute of Medicine. Crossing the Quality Chasm: A New Health System for the 21st Century.

[8] Bruce J. (1990). Fundamental elements of the quality of care: a simple framework. Stud Fam Plann.

[9] Gavin L., Moskosky S., Carter M., Curtis K., Glass E., Godfrey E. (2014). Providing quality family planning services: Recommendations of CDC and the U.S. Office of Population Affairs. MMWR Recomm Rep.

[10] Brook R.H., McGlynn E.A., Cleary P.D. (1996). Quality of health care. Part 2: measuring quality of care. N Engl J Med.

[11] Donabedian A. (1966). Evaluating the quality of medical care. Milbank Mem Fund Q.

[12] Gakidou E., Vayena E. (2007). Use of modern contraception by the poor is falling behind. PLoS Med.

[13] National Population Council, Mexico. National Population Program 2008–2012. Demographic change and development [in Spanish]. http://www.conapo.gob.mx/work/models/CONAPO/Resource/385/1/images/PNP_2008_2012.pdf. Published 2012. Accessed March 8, 2016.

[14] Sedgh G., Finer L.B., Bankole A., Eilers M.A., Singh S. (2015). Adolescent pregnancy, birth, and abortion rates across countries: levels and recent trends. J Adolesc Health.

[15] Allen-Leigh B., Villalobos-Hernández A., Hernández-Serrato M.I., Suárez L., de la Vara E., de Castro F. (2013). Use of contraception and family planning in adolescent and adult women in Mexico [in Spanish]. Salud Publica Mex.

[16] Darney B.G., Weaver M.R., Sosa-Rubi S.G., Walker D., Servan-Mori E., Prager S. (2013). The Oportunidades conditional cash transfer program: effects on pregnancy and contraceptive use among young rural women in Mexico. Int Perspect Sex Reprod Health.

[17] Nair M., Baltag V., Bose K., Boschi-Pinto C., Lambrechts T., Mathai M. (2015). Improving the Quality of Health Care Services for Adolescents, Globally: A Standards-Driven Approach. J Adolesc Health.

[18] National Institute of Statistics and Geography. National Survey of Dynamic Demography 2009 [in Spanish]. http://internet.contenidos.inegi.org.mx/contenidos/productos//prod_serv/contenidos/espanol/bvinegi/productos/encuestas/hogares/enadid/enadid2009/ENADID_2009_Pan_Soc.pdf. Published 2011. Accessed December 10, 2015.

[19] National Institute of Statistics and Geography. National Survey of Dynamic Demography 2014 [in Spanish]. http://internet.contenidos.inegi.org.mx/contenidos/productos//prod_serv/contenidos/espanol/bvinegi/productos/nueva_estruc/702825075255.pdf. Published 2014. Accessed December 10, 2015.

[20] National Population Council. National Survey of Dynamic Demography 2006 [in Spanish]. http://www.conapo.gob.mx/work/models/CONAPO/Enadid2006/docs/Reporte%20Final%20ENADID%202006.pdf. Published 2008. Accessed December 10, 2015.

[21] Meuwissen L.E., Gorter A.C., Knottnerus J.A. (2006). Perceived quality of reproductive care for girls in a competitive voucher programme. A quasi-experimental intervention study, Managua, Nicaragua. International J Qual Health Care.

[22] Tumlinson K., Pence B.W., Curtis S.L., Marshall S.W., Speizer I.S. (2015). Quality of Care and Contraceptive Use in Urban Kenya. Int Perspect Sex Reprod Health.

[23] Heiby J.R., Armbruster D., Jacobs T.A. (2014). Better care for every patient, every time: improving quality in low health systems. BJOG.

[24] World Health Organization (2006). Quality of care: a process for making strategic choices in health systems.

[25] National Population Council. National Strategy for the Prevention of Teen Pregnancy. http://www.conapo.gob.mx/work/models/CONAPO/Resource/2441/1/images/ENAPEA_V10.pdf. Published 2014. Accessed March 3, 2016.

[26] Nguyen H., Snider J., Ravishankar N., Magvanjav O. (2011). Assessing public and private sector contributions in reproductive health financing and utilization for six sub-Saharan African countries. Reprod Health Matters.

[27] Hong R., Montana L., Mishra V. (2006). Family planning services quality as a determinant of use of IUD in Egypt. BMC Health Serv Res.

