# Supplementary material for: Comparison of family-planning service quality reported by adolescents and young adult women in Mexico
Source: Int J Gynaecol Obstet. 2016 Jul;134(1):22–8. doi: 10.1016/j.ijgo.2015.12.003 (PMC4925379; doi:10.1016/j.ijgo.2015.12.003)
Supplement: Supplementary material S1 — Patient-reported items of the quality of familyplanning services items. [file mmc1.docx]

Before you were prescribed [current method]:

Technical quality

- Were you told about other methods you could use?
- Were possible side effects explained to you?
- Were you told to come back in the case of side effects?

Interpersonal quality

- Were you given enough time for all the information you needed?
- Were your doubts about the method addressed?
