# Supplementary material for: Comparison of family-planning service quality reported by adolescents and young adult women in Mexico
Source: Int J Gynaecol Obstet. 2016 Jul;134(1):22–8. doi: 10.1016/j.ijgo.2015.12.003 (PMC4925379; doi:10.1016/j.ijgo.2015.12.003)
Supplement: Supplementary material S2 — Missing survey data. [file mmc2.docx]

| Variable | Missing data, % |
| --- | --- |
| Individual characteristics |  |
| Age | 0.00 |
| Educational gap | 0.17 |
| Married or co-habiting exposition | 0.00 |
| Currently working | 0.04 |
| Number of children born alive | 6.40 |
| Health insurance | 1.79 |
| Household characteristics |  |
| Head of household speaks an indigenous language | 0.00 |
| Man as head of household | 0.00 |
| Household size 6 people or greater | 0.00 |
| Household wealth quintile | 8.42 |
| Municipality size | 0.00 |
| State | 0.00 |
